# Social inequalities in a population based colorectal cancer screening programme in the Basque Country

**DOI:** 10.1186/s12889-015-2370-5

**Published:** 2015-10-05

**Authors:** Jose Luis Hurtado, Amaia Bacigalupe, Montse Calvo, Santi Esnaola, Nere Mendizabal, Isabel Portillo, Isabel Idigoras, Eduardo Millán, Eunate Arana-Arri

**Affiliations:** Araba County, Osakidetza-Basque Health Service, Araba, Spain; Department of Sociology 2, University of the Basque Country (UPV/EHU), Bizkaia, Spain; Directorate of Health Planning, Department of Health, Basque Government, Araba, Spain; Primary Care Research Unit, Bizkaia, Spain; Colorectal Cancer Screening Programme Coordinating Centre, Basque Health Service, Bizkaia, Spain; Healthcare Services Sub-directorate, Osakidetza-Basque Health Service, Araba, Spain; Clinical Epidemiology Unit, Cruces University Hospital, BioCruces Health Research Institute, 48903 Barakaldo-Bizkaia, Spain

**Keywords:** Social inequalities, Colorectal cancer, Screening programme

## Abstract

**Background:**

While it is known that a variety of factors (biological, behavioural and interventional) play a major role in the health of individuals and populations, the importance of the role of social determinants is less clear. The effect of social inequality on population-based screening for colorectal cancer (CRC) could limit the value of such programmes. The present study aims to determine whether such inequalities exist.

**Methods:**

Data was obtained from the population-based screening programme administered in the Autonomous Community of the Basque Country, Spain, with a target population aged 50 to 69, first invited to participate between 2009 and 2011. The magnitude of inequality was analysed using the odds ratio (taking the least disadvantaged socioeconomic quintile as the reference population), the population attributable risk and the relative index of inequality, based on the regression, which is the ratio of the rates in the most and least disadvantaged socioeconomic groups.

**Results:**

The target population comprised 242,394 people, with the test kit successfully sent to 95.1 % (230,510). The overall response rate was 64.3 % (67.1 in women and 61.4 % men).

Among women, the highest participation was in the third quintile (71.5 %) and the lowest in the first – the least disadvantaged (65.7 %). The lowest and highest rates of people with identified lesions were in the second and fourth quintiles (14.7/1000 and 17.0/1000 respectively).

Among men, the response rate was lowest in the fifth – most disadvantaged – quintile (60.2 %). The highest rate of identified lesions was in the fifth quintile; 38 % higher than the first (55.7/1000 compared to 41.0/1000).

**Conclusions:**

Sex and socioeconomic group influence the rate of participation in the CRC programme and the rate of lesions found in the participants.

Any public health programme is morally and ethically obliged to strive for equity and effectiveness. Improving participation of men and socially disadvantaged groups should be taken in account.

## Background

The health of individuals and populations depends on a wide range of factors, including biological variables, health-related behaviour and health system performance. There is growing evidence, however, that social determinants of health play a highly important role [1, 2]. The uneven distribution of these determinants according to different social stratification criteria - social class, educational level, degree of deprivation of area of residence, etc. - generates health inequalities, with those belonging to more disadvantaged socioeconomic groups or living in areas of greater social deprivation consistently evidencing worse health indicators and unhealthier lifestyles and habits [3–5].

The WHO Commission on Social Determinants of Health (CSDH) said in its 2008 report [1] that the organization and characteristics of health systems also play an important role in health equity, either reducing inequalities generated by other social determinants or, conversely, amplifying them. The “inverse care law”, according to which the availability of health care tends to vary inversely with the need of the target population [6, 7], is a well-identified mechanism for explaining the amplification phenomenon. Despite their universal approach, population-based disease prevention and health promotion programmes implemented by health authorities do not always guarantee equal access for and impact on the various social groups, which can lead to a worsening of social inequalities in health [8]. Some postulate reduced responsiveness to disease prevention and health promotion messages among people living in disadvantaged socioeconomic areas - due to competition from or prioritization of more essential needs - while others identify reduced availability and implementation of programmes in such contexts [9].

Specifically, health-system-driven population screening programmes (including population-based CRC screening) help decrease the impact of certain diseases or health problems on the population, through early detection. The aim is to reduce the incidence of progressive disease and related mortality, which are high in all developed countries. According to WHO estimates CRC affected over 471.240 people in 2012, with almost 228.275 dying from the disease in the European Union. In Spain, CRC is in first place for incidence (32,240 people) and takes second place for mortality (14,700 deaths), outnumbered only by deaths from lung cancer [10]. Basque Country data follows the same trend, with a significant increase in incidence in the last two decades [11].

The ability of screening to reduce CRC mortality depends heavily on the degree of participation in the population, but also on the chosen screening method. A faecal occult blood test (FOBT) performed every two years can reduce mortality by 19 %, whereas first-line colonoscopy offers a 68 % reduction [12]. The guaiac-based FOBT has now been replaced by the faecal immunochemical test (FIT), thus increasing test sensitivity, and colonoscopy has improved in terms of equipment, training and quality assurance [13]. In Spain, according to National Cancer Strategy, FOBT every two years is recommended for 50–69 years old population and colonoscopy as a confirmatory test in positive cases, being included as a basic service for all population in 2014 [14].

With regard to participation, the literature shows that even in well-established programmes with high population coverage, significant social inequalities exist, by socioeconomic status, gender, age and ethnicity [15–22]. Women (perhaps due to their greater awareness of the importance of self-care, as well as their role as the household's main caregiver) and older people (>60) show the highest screening rates [15–17], while men evidence greater participation in invasive tests [18]. Most studies agree that the main causes of non-participation among the most disadvantaged socioeconomic groups are: lack of information about the disease, prioritization of other problems with a greater impact on everyday life, and not understanding the written communications that arrive in the post [15, 16].

In 2013, 11 of Spain's 17 regions had population-based CRC screening programmes in place, which, when combined, covered 20 % of the Spanish population aged between 50 and 69 [23]. The Basque Country was the region with the greatest coverage (97.9 %) in 2013, combined with high participation (64.3 %) [24], being their target population around 583,000 people, which 51.4 % are women. This can be attributed to a Primary Care programme that began in 2009, based on the use of a two-yearly FIT, with those patients that returned a positive FIT referred to the public hospital for colonoscopy under sedation, in order to confirm the diagnosis. A strategy of home delivery of testing kits combined with provision of a broad time band for delivering the samples to health centres was implemented to facilitate participation, as well as detailed programme information and access to a freephone information service. However, the potential existence of social inequalities in the various phases of screening may limit the effectiveness of the programme and bring the “inverse care law” into play. The aim of this article is thus to describe the magnitude of social inequalities in population-based CRC screening in the Basque Country between 2009 and 2011, according to the level of socioeconomic deprivation of the area of residence, focusing mainly on response rates and lesions identified. This data was reported with regard to sex in a previous publication [25]. Nowadays, the programme is continuing rolling-out with the same criteria inviting progressively in successive rounds all the target population.

## Methods

This is a cross-sectional study of people aged 50 to 69 years invited to participate for the first time in the Basque Country’s CRC screening programme between 2009 and 2011. The study was approved by the Euskadi Ethics Committees and each participant provided written informed consent.

### Study subjects

People aged 50 to 69, living in the Basque Country and registered with one of the 60 health centres in which the CRC screening programme was implemented between 2009 and 2011, equivalent to about 50 % of the region's population in the given age range. The population covered by the programme at that time was not complete because of the colonoscopy capacity in hospitals was limited then. Exclusion criteria were: being under surveillance for previously diagnosed CRC, having a high-risk family history of CRC, or having colonoscopy/sigmoidoscopy follow-up for adenoma during the previous 5 years, or total colectomy or terminal/irreversible disease or unknown address.

### Study variables

#### Successfully-invited population

People aged 50–69 years meeting no exclusion criteria, who were sent a letter of invitation that was not returned due to the address being incorrect.

#### Participant

Of all those who were successfully invited, those who handed in the kit and for whom a correct result was returned (negative/positive).

#### Positive FIT test

20 ng/ml according to manufacturer's instructions. OC-Sensor (2009–2011) and Sentinel (2009–2010) were used.

*People with premalignant lesion*, defined as the discovery after colonoscopy of advanced (medium- and high-risk) adenomas, as defined in the 2010 European Guidelines [26] and *malignant lesion* invasive carcinoma (≥pT1) according to the pathology report in the patient record.

### Source of data

#### Socioeconomic status

Each study participant was assigned the socioeconomic deprivation index (DI) of their small area of residence. This composite index was calculated by the Basque Government Health Department's Health Research Service, using the Medea Project [26] criteria, from simple indicators in the 2001 Census: unemployment, manual workers, casual workers, insufficient education and insufficient education among young people. The DI was divided into quintiles, with the first being the least disadvantaged and the fifth the most disadvantaged. Participant data was linked to the DI variable using the Individual Health Card code, or the corporative identification code, in those cases where the participant did not have an Individual Health Card number. The DI was successfully assigned to 95.1 % of participants, while address information quality did not permit to link the remaining 4.9 %.

All data was obtained from the Basque Country's population-based CRC screening programme database, which is linked to patient records. This allows all cases to be followed, from submission of the sample, through analysis, colonoscopy, pathology and follow-up.

### Statistical analysis

Age-standardized rates were calculated using the direct method for the different variables, for total participants and by level of deprivation, using Basque Country residents between 50 and 69 years in 2011 as the reference population. To obtain participation rates, the number of people who participated was divided by the *successfully*-*invited population*. To obtain lesion rates, the number of people in whom lesions were detected was divided by the total *participant* population. To obtain malignant lesion rates, the number of people in whom malignant lesions were detected was divided by the total *participant* population. To obtain the positive predictive value (PPV), the number of people with lesions detected was divided by the number of people with positive FIT results. The comparative analysis between men and women was performed using indirectly standardized ratios and between age groups, using odds ratios estimated by logistic regression models. The magnitude of inequalities was analysed using: (a) odds ratios estimated by logistic regression models, taking as the reference population the least disadvantaged socioeconomic quintile, (b) the population-attributable risk (PAR) and (c) the relative index of inequality (RII). RII is based on the regression and consists of the rate ratio between the most and least disadvantaged groups respectively. These results were estimated using logistic regression models. All analyses were performed separately for men and women. P value less than 0.05 was considered statistically significant using 2-sided test. The analysis was performed with R v. 2.13.1.

## Results

**Fig. 1:**
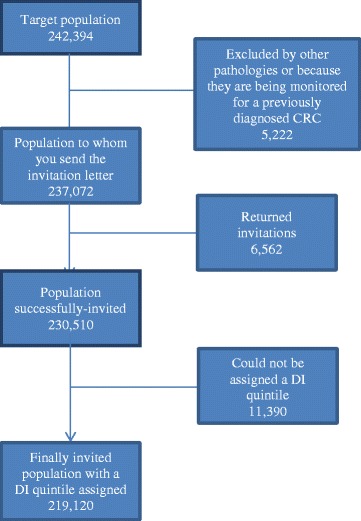
Flowchart

Between 2009 and 2011, the target population comprised 242,394 people (50.9 % women), with the test kit successfully sent to 230,510 (95.1 %). Of this group, a DI was successfully assigned to 219,120 people (95.1 %) (Fig. 1).

The overall response rate was 64.3 % (67.1 in women and 61.4 % men). In 6.7 % of participants, the FIT result was positive (4.8 of women and 9.0 % of men). Of these, 92.6 % were given a colonoscopy (92.0 of women and 93.0 % of men), resulting in the detection of 4,523 lesions, of which 3,952 were premalignant and 571 were carcinomas, with 383 in stages I-II (Table 1).

**Table 1:** Baseline table with data and distribution percentages

	Totals	%	Women	%	Men	%
Population	242,394		122,901		119,493	
age (years):						
50-54	81,605	33.7	41,065	33.4	40,540	33.9
55-59	59,633	24.6	30,130	24.5	29,503	24.7
60-64	61,266	25.3	31,176	25.4	30,090	25.2
65-69	39,890	16.5	20,530	16.7	19,360	16.2
Successfully-invited population, out of the total population	230,510	97.2	117,573	97.6	112,937	96.8
Participants, out of the successfully-invited population	148,265	64.3	78,916	67.1	69,349	61.4
FOBT results for participants						
Positive	9,961	6.7	3,751	4.8	6,210	9.0
Negative	138,165	93.2	75,091	95.2	63,074	91.0
Error	130	0.1	71	0.1	59	0.1
Lost	9	0.0	3	0.0	6	0.0
colonoscopy performed after positive FOBT						
No	735	7.4	300	8.0	435	7.0
Yes	9,226	92.6	3,451	92.0	5,775	93.0
Colonoscopy results						
Negative	4,639	50.3	2211	64.1	2,428	42.0
N/A or inconclusive	64	0.7	20	0.6	44	0.8
Premalignant or malignant lesion	4,523	49.0	1220	35.4	3,303	57.2
Type of premalignant or malignant lesion:						
Premalignant	3,952	87.4	1,034	84.8	2,918	88.3
Cancer	571	12.6	186	15.2	385	11.7
Cancer results:						
stage I/II	383	67.1	118	63.4	265	68.8
stage lII/IV	181	31.7	65	34.9	116	30.1
stage unknown / N/A	6	1.1	2	1.1	4	1.0
stage lost	1	0.2	1	0.5	0	0.0

Among participants, the standardized rate of people with lesions was 48.5/1000 in men, three times higher than the 15.7/1000 for women. The rate of people with malignant lesions was also 2.34 times higher in men than women (5.49/1000 vs. 2.42/1000). The PPV of the test was 52.2 % in men compared to 32.5 % in women (Table 2).

**Table 2:** Standardized rates and standardized rate ratio between sexes

	Women		Men			
Indicators	standardized rates^a^	(95 % CI)	standardized rates^a^	(95 % CI)	RR^b^	(95 % CI)
Participants from successfully-invited population	67.14	(66.67-67.62)	61.69	(61.23-62.16)	0.91	(0.91-0.92)
Lesions among participants	15.69	(14.82-16.61)	48.50	(46.84-50.20)	3.07	(2.96-3.17)
Cancer among participants	2.42	(2.08-2.80)	5.79	(5.22-6.41)	2.34	(2.12-2.58)
Stage III/IV cancer among participants	0.82	(0.63-1.05)	1.76	(1.45-2.12)	2.03	(1.69-2.43)
Colonoscopies performed after + FOBT	92.07	(88.99-95.23)	92.95	(90.52-95.44)	1.01	(0.99-1.04)
Lesions identified among colonoscopy patients	35.30	(33.33-37.36)	56.11	(54.17-58.10)	1.62	(1.56-1.67)
Lesions, out of those with + FOBT	32.50	(30.68-34.40)	52.19	(50.39-54.04)	1.63	(1.58-1.69)

^a^Rates per 1000 for lesions, cancer and cancer stage; percentage for participants, colonoscopy after + FOBT and colonoscopy patients with lesions

^b^Standardized rate ratio for men/women

Both men and women aged 60–64 participated most in the programme, while those between 50–54 ages participated least. Lesion detection rates and people with malignant rates were higher in the older age groups. The rate of people with cancer in stages III and IV only increased with age in men (*p* < 0.001).

Among men, the PPV of the test increased with age, ranging from 43.60 % to 59.67 %. Among women, the lowest and the highest PPV were 31.62 % (50–54) and 33.01 (65–69), respectively.

The proportion of people who underwent a colonoscopy following a positive FIT result was not associated with age (Table 3).

**Table 3:** PPV of the test, participation, lesion and colonoscopy rates following positive test result, by age group

Women						Men					
Age groups	successfully-invited population	no. of participants	% participation	OR	(95 % CI)	Age groups	successfully-invited population	no. of participants	% participation	OR	(95 % CI)
50-54	37379	24609	65.84	1.00		50-54	36221	21033	58.07	1.00	
55-59	27637	19375	70.11	1.22	(1.18-1.26)	55-59	26324	16692	63.41	1.25	(1.21-1.29)
60-64	28760	20434	71.05	1.27	(1.23-1.32)	60-64	26866	18144	67.54	1.50	(1.45-1.55)
64-69	18758	12635	67.36	1.07	(1.03-1.11)	64-69	17175	11457	66.71	1.45	(1.40-1.51)
				*p* < 0.001						*p* < 0.001	
Age groups	no. of participants	no. of lesions (premal. + malignant)	rate of lesions (premal. + malignant) among participants^a^	OR	(95 % CI)	Age groups	no. of participants	no. of lesions (premal. + malignant)	rate of lesions (premal. + malignant) among participants	OR	(95 % CI)
50-54	24609	302	12.27	1.00		50-54	21033	593	28.19	1.00	
55-59	19375	273	14.09	1.15	(0.97-1.35)	55-59	16692	777	46.55	1.7	(1.51-1.88)
60-64	20434	357	17.47	1.43	(1.22-1.66)	60-64	18144	1058	58.31	2.1	(1.93-2.37)
64-69	12635	257	20.34	1.66	(1.41-1.97)	64-69	11457	799	69.74	2.6	(2.31-2.87)
				*p* < 0.001						*p* < 0.001	
Age groups	no. of participants	no. of malignant lesions	rate of malignant lesions among participants^a^	OR	(95 % CI)	Age groups	no. of participants	no. of malignant lesions	rate of malignant lesions among participants	OR	(95 % CI)
50-54	24609	38	1.54	1.00		50-54	21033	47	2.23	1.00	
55-59	19375	37	1.91	1.24	(0.78-1.95)	55-59	16692	77	4.61	2.07	(1.45-3.00)
60-64	20434	59	2.89	1.87	(1.25-2.83)	60-64	18144	119	6.56	2.97	(2.13-4.20)
64-69	12635	47	3.72	2.40	(1.56-3.70)	64-69	11457	126	11.00	5.02	(3.62-7.10)
				*p* < 0.001						*p* < 0.001	
Age groups	no. of participants	no. of carcinomas stage III/IV	no. of carcinomas stage III/IV	OR	(95 % CI)	Age groups	no. of participants	no. of carcinomas stage III/IV	no. of carcinomas stage III/IV	OR	(95 % CI)
50-54	24609	16	0.65	1.00		50-54	21033	10	0.48	1.00	
55-59	19375	15	0.77	1.19	(0.58-2.41)	55-59	16692	19	1.14	2.40	(1.14-5.37)
60-64	20434	22	1.08	1.65	(0.87-3.19)	60-64	18144	37	2.04	4.33	(2.24-9.20)
64-69	12635	10	0.79	1.20	(0.52-2.61)	64-69	11457	44	3.84	8.23	(4.32-17.34)
				*p* = 0.488						*p* < 0.001	
Age groups	people with + FOBT	people who had colonoscopy	% colonoscopies	OR	(95 % CI)	Age groups	people with + FOBT	people who had colonoscopy	% colonoscopies	OR	(95 % CI)
50-54	955	881	92.25	1.00		50-54	1360	1259	92.57	1.00	
55-59	827	773	93.47	1.17	(0.82-1.69)	55-59	1458	1363	93.48	1.17	(0.87-1.57)
60-64	1083	991	91.51	0.90	(0.65-1.23)	60-64	1871	1739	92.94	1.06	(0.81-1.39)
64-69	785	720	91.72	0.91	(0.64-1.29)	64-69	1339	1266	94.55	1.40	(1.03-1.92)
				*p* = 0.446						*p* = 0.150	
Age groups	people who had colonoscopy	no. of lesions (premal. + malignant)	% lesions found in colonoscopy	OR	(95 % CI)	Age groups	people who had colonoscopy	no. of lesions (premal. + malignant)	% lesions found in colonoscopy	OR	(95 % CI)
50-54	881	302	34.28	1.00		50-54	1259	593	47.10	1.00	
55-59	773	273	35.32	1.05	(0.85-1.28)	55-59	1363	777	57.01	1.49	(1.27-1.73)
60-64	991	357	36.02	1.08	(0.89-1.30)	60-64	1739	1058	60.84	1.75	(1.51-2.02)
64-69	720	257	35.69	1.07	(0.87-1.31)	64-69	1266	799	63.11	1.92	(1.64-2.25)
				*p* = 0.883						*p* < 0.001	
Age groups	people with + FOBT	no. of lesions (premal. + malignant)	PPV	OR	(95 % CI)	Age groups	people with + FOBT	no. of lesions (premal. + malignant)	PPV	OR	(95 % CI)
50-54	955	302	31.62	1.00		50-54	1360	593	43.60	1.00	
55-59	827	273	33.01	1.06	(0.87-1.30)	55-59	1458	777	53.29	1.48	(1.27-1.71)
60-64	1083	357	32.96	1.06	(0.88-1.28)	60-64	1871	1058	56.55	1.69	(1.46-1.94)
64-69	785	257	32.74	1.06	(0.86-1.29)	64-69	1339	799	59.67	1.91	(1.64-2.23)
				*p* = 0.919						*p* < 0.001	

*OR* Odds ratios adjusted by DI quintile, *CI* Confidence interval; p: significance value of the likelihood ratio test for the association between age and outcome variable

^a^rates per 1000 inhabitants

By socioeconomic status, among women, the highest percentage participation was in the third quintile (71.5 %) and the lowest participation was in the - least disadvantaged - first quintile (65.7 %). This quintile also underwent the lowest colonoscopy rate following a positive FIT result (89.1 %). The lowest and highest rates of people with lesions identified were in the second and fourth quintiles respectively (14.7/1000 compared to 17.00/1000), although overall the effect of socioeconomic status was not statistically significant (*p* =0.600). Neither did the DI show any statistically significant association with the rate of people with malignant lesions (*p* = 0.824), rate of people with cancer in stages III and IV (*p* = 0.853), or the PPV (*p* = 0.197). No RII was calculated in women, since on reviewing the tables; no linear association was visible between the DI and the results (Table 4).

**Table 4:** PPV of the test, standardized participation, lesion and colonoscopy following positive test result, by socioeconomic stratum of place of residence

Women						Men					
DI quintile	successfully-invited population	no. of participants	% participation	OR	(95 % CI)	DI quintile	successfully-invited population	no. of participants	% participation	OR	(95 % CI)
I (least disadvantaged)	26193	17228	65.73	1.00		I (least disadvantaged)	23947	14743	61.87	1.00	
II	24373	17026	69.89	1.21	(1.16-1.25)	II	23373	15079	64.91	1.14	(1.10-1.19)
III	22632	16160	71.46	1.30	(1.25-1.35)	III	21440	14086	66.07	1.20	(1.15-1.25)
IV	19927	13819	69.30	1.17	(1.13-1.22)	IV	19343	12349	64.07	1.10	(1.06-1.14)
V (most disadvantaged)	19409	12820	66.06	1.01	(0.97-1.05)	V (most disadvantaged)	18483	11069	60.25	0.93	(0.90-0.97)
				*p* < 0.001						*p* < 0.001	
DI quintile	no. of participants	no. of lesions (premal. + malignant)	rate of lesions (premal. + malignant) among participants^a^	OR	(95 % CI)	DI quintile	no. of participants	no. of lesions (premal. + malignant)	rate of lesions (premal. + malignant) among participants	OR	(95 % CI)
I (least disadvantaged)	17228	255	15.39	1.00		I (least disadvantaged)	14743	594	40.99	1.00	
II	17026	246	14.61	0.97	(0.81-1.16)	II	15079	705	48.73	1.18	(1.06-1.32)
III	16160	258	16.12	1.07	(0.90-1.27)	III	14086	703	50.76	1.25	(1.12-1.40)
IV	13819	233	16.99	1.11	(0.93-1.33)	IV	12349	617	49.83	1.23	(1.10-1.38)
V (most disadvantaged)	12820	197	15.40	1.01	(0.84-1.22)	V (most disadvantaged)	11069	608	55.68	1.38	(1.23-1.55)
				*p* = 0.600						*p* = 0.002	RII 1.37 (1.21-1-55)
DI quintile	no. of participants	no. of malignant lesions	rate of malignant lesions among participants^a^	OR	(95 % CI)	DI quintile	no. of participants	no. of malignant lesions	rate of malignant lesions among participants	OR	(95 % CI)
I (least disadvantaged)	17228	38	2,25	1,00		I (least disadvantaged)	14743	78	5,46	1,00	
II	17026	37	2,24	0,98	(0.62-1.54)	II	15079	85	6,14	1,08	(0.80-1.48)
III	16160	38	2,37	1,05	(0.67-1.65)	III	14086	93	6,89	1,25	(0.92-1.69)
IV	13819	31	2,35	0,97	(0.60-1.56)	IV	12349	45	3,60	0,66	(0.45-0.95)
V (most disadvantaged)	12820	37	2,90	1,25	(0.79-1.97)	V (most disadvantaged)	11069	68	6,37	1,14	(0.82-1.58)
				*p* = 0.824						*p* = 0.006	
DI quintile	no. of participants	no. of carcinomas stage III/IV	no. of carcinomas stage III/IV	OR	(95 % CI)	DI quintile	no. of participants	no. of carcinomas stage III/IV	no. of carcinomas stage III/IV	OR	(95 % CI)
I (least disadvantaged)	17228	10	0.70	1.00		I (least disadvantaged)	14743	24	1.97	1.00	
II	17026	14	0.84	1.27	(0.59-2.76)	II	15079	27	2.03	1.00	(0.59-1.69)
III	16160	10	0.67	0.97	(0.42-2.22)	III	14086	20	1.52	0.75	(0.41-1.32)
IV	13819	12	0.89	1.23	(0.54-2.76)	IV	12349	12	0.94	0.49	(0.24-0.93)
V (most disadvantaged)	12820	13	0.99	1.44	(0.65-3.20)	V (most disadvantaged)	11069	22	2.13	1.02	(0.58-1.77)
				*p* = 0.853						*p* = 0.137	
DI quintile	people with + FOBT	people who had colonoscopy	% colonoscopies	OR	(95 % CI)	DI quintile	people with + FOBT	people who had colonoscopy	% colonoscopies	OR	(95 % CI)
I (least disadvantaged)	773	688	89.07	1.00		I (least disadvantaged)	1168	1056	90.51	1.00	
II	789	723	91.64	1.34	(0.96-1.89)	II	1295	1205	92.96	1.41	(1.06-1.89)
III	735	685	93.45	1.68	(1.17-2.44)	III	1292	1227	94.89	2.01	(1.47-2.77)
IV	688	647	93.99	1.94	(1.32-2.88)	IV	1154	1102	95.60	2.25	(1.61-3.18)
V (most disadvantaged)	665	622	93.65	1.78	(1.22-2.63)	V (most disadvantaged)	1119	1037	92.51	1.33	(0.99-1.80)
				*p* = 0.003						*p* = <0.001	
DI quintile	people who had colonoscopy	no. of lesions (premal. + malignant)	% lesions found in colonoscopy	OR	(95 % CI)	DI quintile	people who had colonoscopy	no. of lesions (premal. + malignant)	% lesions found in colonoscopy	OR	(95 % CI)
I (least disadvantaged)	688	255	37.01	1.00		I (least disadvantaged)	1056	594	55.46	1.00	
II	723	246	33.88	0.88	(0.70-1.09)	II	1205	705	57.23	1.10	(0.93-1.30)
III	685	258	37.72	1.03	(0.82-1.27)	III	1227	703	56.27	1.05	(0.88-1.24)
IV	647	233	36.14	0.95	(0.76-1.19)	IV	1102	617	54.97	0.99	(0.83-1.17)
V (most disadvantaged)	622	197	31.01	0.79	(0.63-0.99)	V (most disadvantaged)	1037	608	57.61	1.09	(0.91-1.30)
				*p* = 0.143						*p* = 0.669	
DI quintile	people with + FOBT	no. of lesions (premal. + malignant)	PPV	OR	(95 % CI)	DI quintile	people with + FOBT	no. of lesions (premal. + malignant)	PPV	OR	(95 % CI)
I (least disadvantaged)	773	255	32.97	1.00		I (least disadvantaged)	1168	594	50.18	1.00	
II	789	246	31.05	0.92	(0.74-1.14)	II	1295	705	53.25	1.16	(0.99-1.36)
III	735	258	35.26	1.10	(0.89-1.36)	III	1292	703	53.49	1.16	(0.99-1.36)
IV	688	233	33.98	1.04	(0.83-1.29)	IV	1154	617	52.57	1.11	(0.94-1.31)
V (most disadvantaged)	665	197	29.00	0.85	(0.68-1.07)	V (most disadvantaged)	1119	608	53.29	1.14	(0.96-1.34)
				*p* = 0.197						*p* = 0.349	

*OR* Age-adjusted odds ratios*, CI* Confidence interval

DI quintile: Socioeconomic deprivation index quintile proposed by the MEDEA project; p: significance value of the likelihood ratio test for the association between DI and outcome variable; RII: relative index of inequality

^a^age-standardized rates per 1000 inhabitants (reference population Basque Country 2011)

The PAR or percentage of preventable lesions if the total rates had been those of the DI quintile with the lowest lesion rate is 6.7 % in women, representing 81 fewer lesions if the rate of people with lesions for the whole population had been that of the second quintile.

Among men, the participation rate was significantly lower in the fifth – most disadvantaged – quintile (60.2 %) compared to other quintiles. The first quintile had the smallest proportion of people who underwent a colonoscopy after a positive result (90.5 %), followed by the fifth quintile (92.5 %), with the fourth quintile showing the largest (95.6 %). The rates of people with lesions identified were highest in the fifth quintile and were 38 % higher than those in the first quintile (55.7/1000 compared to 41.0/1000). The highest rate of malignant lesions was in the third quintile (6.9/1000), although this was not significantly higher than the reference quintile (OR 1.25, 95 % CI (0.92-1.69)). No significant association was found with the rate of people with cancer in stages III and IV (*p* = 0.137) or with the PPV (*p* = 0.349). The RII for the rate of men with lesions (premalignant and malignant) was 1.37 (95 % CI 1.21-1.55) (Table 4).

The PAR or percentage of preventable lesions in men was 16.0 %, which is equivalent to 529 fewer people with lesions if the overall rate had been that of the DI quintile with the lowest rate (first quintile).

Finally, if a rate similar to that obtained in the quintile with the highest rate of participation (71.5 % of women in the third quintile) had been achieved in all socioeconomic quintiles, 466 people with lesions would have been detected within this period (414 men), of which 56 would have been malignant (48 men).

## Discussion

This study reveals socioeconomic inequalities in a number of quality indicators for the Basque Country's colorectal cancer screening programme between 2009 and 2011.

Men in the most disadvantaged socioeconomic class evidence the lowest participation rate, but also the highest rates of premalignant and malignant lesions, between 23 and 55 % higher than the least disadvantaged quintile. Among women, those in the groups with the lowest and highest socioeconomic status participate the least, and no differences are observed in the rate of people with lesions in the most disadvantaged groups. No differences in PPV are detected between socioeconomic groups, either in men or in women, and neither are there any differences between groups in the percentage of colonoscopies performed after a positive FIT result.

Overall participation rate (64.3 %) is close to the 65 % target set by the 2011 European Guidelines [26], although it is lower in men than in women, and lower in the youngest and oldest groups. A similar pattern is found in other CRC screening programmes [15, 19, 27–29].

Attempting to explain this differential behaviour by sex and age, some studies postulate that men might be less interested about their health, as well as being afraid of the diagnostic test, while women might assume the role of caregiver, leading more of them to worry about their health, for the sake of those around them [15, 16]. The influence of a dominant societal perception of masculinity has been described as an important factor in explaining inferior participation among men, since CRC screening entails the risk of having to undergo an invasive procedure - colonoscopy- that might conflict with normative “male” beliefs [30, 31]. In younger people, feeling healthy and less vulnerable to the disease could be a barrier to participation. Finally not being aware of the importance of screening or not having had their doctor recommend the screening process are some factors that might reduce participation in the older age groups [15, 16]. These hypothetical explanations should be treated with caution, however, since it is widely recognised that knowledge and health beliefs have a limited capacity to explain people's actual behaviour, and underlying motives are particularly difficult to ascertain [32].

The rate of people with premalignant and malignant lesions detected in colonoscopy is three times higher in men than in women. In the older group, it is 2.6 and 1.7 times higher than the younger group, for men and women respectively (5.02 and 2.4 times higher in the older group if we only take into account rates of people with malignant lesions). These figures corroborate the strong association of age and sex with the probability of detecting a premalignant or malignant lesion that has been reported in other studies [33–36].

The PPV for detection of premalignant and malignant lesions increases with age and is significantly higher in men than in women. These patterns are already known and are probably due in large part to differences in prevalence between certain subgroups [37–39].

The proportion of colonoscopy performed following a positive FIT result was not associated with age in either sex. Similar results were found by other authors (36-steel, 44-Dupont-Lucasa). The high compliance of the procedure in all the age groups (above 91.5 %) could have played a role in the lack of association.

In both sexes the socioeconomic groups with the lowest participation were, paradoxically, both the least and the most disadvantaged. The former could be due to greater access to private healthcare among the more privileged social classes. In fact public health services are available for all population, but the least deprived normally contract a private insurance as well, where colonoscopy is offered as an opportunistic screening. As already noted above, in reference to the research by Williams, explaining low participation among the most disadvantaged social classes is not a simple task. Lifestyles are composed mostly of actions performed automatically without forethought, with habits and the pursuit of social distinction being the key factors for explaining health and lifestyle, which accounts for the substantial gap between health knowledge and behaviour [32]. Caution is required, therefore, when assessing specific explanations postulated in other articles, such as greater fear of screening, the perception that it is harder to perform, doubts over whether screening is beneficial, lack of knowledge about the test, difficulty in understanding written information and lack of social support [15, 16, 19, 20].

While the published results of other screening programmes show that a lower proportion of those from more disadvantaged social strata attended for colonoscopy after a positive FIT result, it is not the case here [35, 40–42]. Participation among disadvantaged socioeconomic groups is a sign of good programme implementation. Dupont-Lucas et al., in a study that also showed no socioeconomic differences in the percentage of colonoscopies after a positive test, suggest that the voluntary nature of the programme (which is also the case in the present example) allows those members of disadvantaged social groups who would not have been willing to undergo colonoscopy to opt out [43]. On the contrary, the least deprived quintiles show the lowest percentage of colonoscopy for diagnostic confirmation, possibly because a greater proportion are able to access private clinics, whose data is not available to the current study.

The premalignant and malignant lesion detection rates show an inverse association with socioeconomic status in men but not in women. The influence of socioeconomic status on the incidence of CRC is not clear in the literature. Recent systematic reviews and large prospective studies show mixed results. While it appears that in the United States and Canada, lower socioeconomic status is associated with higher rates of CRC, especially in the proximal colon, the tendency in Europe seems to be in the opposite direction, i.e., lower incidence of CRC in lower socioeconomic strata [44–46]. Studies with a higher incidence of CRC among lower socioeconomic strata or those with less education, point to a higher prevalence of modifiable risk factors associated with CRC such as smoking, excessive alcohol consumption, obesity, low levels of physical activity or non-adherence to a Mediterranean diet, as well as greater psychological stress due to socioeconomic status, which may lead to increased susceptibility to disease in general. Moreover, opportunistic CRC screening, which is performed with greater frequency in higher social classes, leads to a higher percentage of cancers avoided in this group, due to lesions being detected in premalignant phases [45, 47, 48]. For the European studies that show a lower incidence of CRC in the lowest socioeconomic strata, it is argued that in addition to a lesser influence of the differential CRC screening effect in Europe, people also have better dietary habits or adhere more closely to a Mediterranean diet, especially in rural areas and southern countries [45–46].

In the Basque Country, according to the 2013 Basque Health Survey, the proportion of men who are smokers, obese or sedentary increases with decreasing social class or education level. Fruit and vegetable intake is also lower in the lower socioeconomic strata, while it seems that the only risk factor that occurs in greater proportion in the higher strata, is that of dangerously high levels of alcohol consumption [49]. The higher prevalence of risk factors in male members of the most disadvantaged groups in the Basque Country may partly account for the results obtained in this study. But not so in the case of women, where no inverse association between social class and lesion detection exists, findings similar to those published by Oliphant et al. with data from a CRC screening programme in the west of Scotland [50]. The authors of the Scottish study could not provide a clear explanation for this differential behaviour between the sexes, although they raised a number of possible reasons, including the possibility of some differences in risk factors between the sexes, such as excessive alcoholism in men or less physical activity in women. In the present study, the 2013 Basque Health Survey reveals that in the Basque Country while tobacco consumption among men increases as social class decreases, no pattern can be seen in women from the age of 45. Nevertheless, the highest prevalence of other risk factors occurs in the most disadvantaged social groups, meaning that the social inequalities in the screening results cannot be explained through these risk factors alone.

Limitations of this study include the fact that, during the study period (2009–2011), the centres participating in the CRC screening programme were not chosen at random, but by the ability of their corresponding referral hospitals to assume the task of performing colonoscopy for screening purposes. This does not preclude broad representation from all socioeconommic groups, since all three provinces are represented and more than 200,000 eligible individuals were included, representing about 50 % of the target population (not taking exclusion criteria into account), with sufficient numbers of people in all relevant variables. It seems, therefore, that the included target population would have similar differential characteristics to the rest of the hypothetical target population of the Basque Country. Further analysis should nevertheless be performed on the definitive data covering 100 % of the population (2014), to assess whether the behaviour is reproduced throughout the entire target population. Moreover, as mentioned above, the non-participant population would most likely have different clinical, social and cultural characteristics to the participating population, and their inclusion might modify the results of this study. Studies that included this non-participant population would be necessary to clarify this question. Finally, using aggregated census tract data to assign socioeconomic status to each individual may lead to incorrect classification of the socioeconomic level in some cases, altering the results to a greater or lesser extent. Numerous studies have, however, demonstrated the use of socioeconomic data of small areas as an approximation to the socioeconomic status of individuals in order to detect health inequalities [51–54]. The absence of individual socioeconomic data has thus been overcome by allocating to subjects the characteristics of the census tract of residence.

## Conclusions

Gender and socioeconomic inequalities are relevant in CRC screening programmes. Both influence participation in the CRC programme and the number of lesions found.

Any public health programme is morally and ethically obliged to strive for equity and also improve programme effectiveness. Achieving a CRC screening programme that can improve participation of men and socially disadvantaged groups would make the programme more effective, and also more equitable. In this way, the Basque Country authorities have included indicators related to screening inequities in general strategies and annual evaluations. Some initiatives to increase men’s participation have been carrying out involving civil associations, factory managers and Primary Care Centres focused in information improvements (video, web-site, training and open meetings), but others related to tackling root causes of hegemonic masculinity in our societies should also need to develop. Regarding most disadvantaged populations, specific qualitative studies will be necessary, so that their main barriers to participating in the programme are adequately understood.
